# Health anxiety in patients with depression with somatic symptoms and psychodermatological disorders

**DOI:** 10.1192/j.eurpsy.2021.887

**Published:** 2021-08-13

**Authors:** A. Ermusheva, M. Vinogradova, A. Tkhostov, L. Pechnikova

**Affiliations:** 1 Department Of Pedagogy And Medical Psychology, I.M. Sechenov First Moscow State Medical University (Sechenov University), Moscow, Russian Federation; 2 Department Of Neuro- And Pathopsychology, Faculty Of Psychology, Lomonosov Moscow State University, Moscow, Russian Federation

**Keywords:** health anxiety, depression with somatic symptoms, psychodermatological disorders

## Abstract

**Introduction:**

As significance of medically unexplained symptoms increases in general practice it is important to discuss psychopathological comorbidity regarding the impact of health anxiety indicating sufferers excessive care use.

**Objectives:**

To study the impact of health anxiety in depression with somatic symptoms.

**Methods:**

50 patients with depression with somatic symptoms compared to 79 patients with psychodermatological disorders with complaints of pathological skin sensations completed the Hospital Anxiety and Depression Scale (HADS) and the Short Health Anxiety Inventory (SHAI). The Mann-Whitney U-Test was applied. The psychosemantic method “Classification of sensations” was used to differentiate patients’ bodily experience. Factor analysis was performed.

**Results:**

Scores on HADS-anxiety and SHAI were significantly higher in depression (U=645, p=0.009; U=89.5; p=0.036), although there were no significant differences on HADS-depression. Factor analysis showed a polarization of bodily experience categories in depression as the first factor (38% of total variance) included negative emotions with somatic sensations of exhaustion and the second factor (10% of total variance) included pleasant sensations and positive emotions with the negative sign of factor loadings. In psychodermatological disorders the first factor (31% of total variance) was quite similar, however the second factor (12% of total variance) included skin and general somatic sensations illustrating the higher concern with somatic symptoms.

**Conclusions:**

Higher health anxiety in depression with somatic symptoms compared to psychodermatological disorders (more concerned with bodily experience) could be associated with patients’ complaints of emotional state indicating differences in psychological mechanisms. The research was supported by Russian Foundation for Basic Research with the Grant 20-013-00799.

